# Recent Advances in Chiral Schiff Base Compounds in 2023

**DOI:** 10.3390/molecules28247990

**Published:** 2023-12-07

**Authors:** China Takeda, Daisuke Nakane, Takashiro Akitsu

**Affiliations:** Department of Chemistry, Faculty of Science, Tokyo University of Science, 1-3 Kagurazaka, Shinjuku-ku, Tokyo 162-8601, Japan; 1322584@ed.tus.ac.jp (C.T.); a29776@rs.tus.ac.jp (D.N.)

Schiff bases (imine or azomethine –N=CH–), which were first obtained by a German chemist, H. Schiff, in 1864, may be part of a popular group of organic compounds prepared from primary amines and aldehyde or ketone. If you look at their history, they have a classical impression, but are an evergreen field; in recent years, a review article with the title “Artistic Beauty” has been published [1]. In this short review article, we will introduce a small portion of the latest research examples and trends, focusing on papers published in 2023, according to typical research theme classifications.

Typical research theme classifications, for example, are presented in this paper.

**(1)**  **Basic (Physical and Chemical) Properties of Schiff Bases**

The stable introduction of optically active moieties that can be applied to organic synthesis, etc. (optically active polycyclic aromatic Schiff bases [2]), and further progress in instrumental analysis and theoretical calculations [3] of these complexes (multiple analyses, the use of tools [4]) is shown. Schiff bases have long been a hot topic, not only in organic compounds, but also in metal complexes, and the study of their stereochemistry continues to be important.

**(2)**  **Formation Mechanism or Principle in the Synthesis of Schiff Bases**

The synthesis of functional groups (C=N) and its mechanism can be established. As a method for introducing optical activity, there is a preferential enrichment of enantiomers through racemic crystallization. A new example from the amino acid Schiff base was shown [5]. Reports of new cases of rare phenomena are expected to play a role in deepening new understanding of their mechanisms and principles.

**(3)**  **Reactions Involving Schiff Bases or Their Metal Complexes**

They are used exclusively as catalysts in reactions, including both organic substances [6,7] and metal complexes [8,9,10]. A summary of the paper title exemplifies the target response of the latest report.

Synthesis and the catalytic activity of bifunctional phase transfer organic catalysts based on camphor [6].

Synthesis of cyclic amino acid using asymmetric phase transfer catalyst [7].

Spectroscopic characterization, catalytic activity, and biological activity of vanadium(V) oxide complexes with chiral tetradentate Schiff bases [8].

New oxidatively stable ligands for the chiral functionalization of amino acids in Ni(II)–Schiff base complexes [9].

Picolinaldehyde–zinc(II)–palladium (0) catalyst system for the asymmetric α-allylation of N-unprotected amino esters [10].

**(4)**  **Schiff Base Ligands in Metal Complexes**

Schiff base metal complexes as reaction catalysts and solid-state physical properties (materials science) have been transferred to (3) and (7), respectively. The molecular design of organic ligands is important for the adsorption of gas molecules onto porous metal-organic complexes [11] and magnetic complexes [12] that respond to external fields, and the ease of synthesis of Schiff bases is utilized. (Actually, the cited references in this short text are limited to journals that can be viewed at the author’s affiliated institution.) Coincidentally, all of these examples are notable for their theme of optical activity, such as the resolution of racemates.

**(5)**  **Analysis or Classification Using Schiff Base Compounds**

Circularly polarized light emission of organic molecules (strictly, typical element complexes) as Schiff base compounds [13], and optically active Raman spectroscopy of biologically related molecules [14], as well as Schiff base metal complexes with polymeric ligands, are employed. There are some reports of phosphorescence [15]. Significant progress has been made in the spectroscopic analysis of new optical activities that go beyond UV–visible circular dichroism. In wet analysis, a highly selective and sensitive ion recognition probe based on a “chiral” thiourea Schiff base has been reported [16]; the study of optical activity is still important.

**(6)**  **Medical or Biological Applications of Schiff Bases**

Spectroscopic studies and confocal and live-cell imaging using chiral Schiff bases as probes were reported [17]. Optically active Schiff bases are also used for imaging, which is also the analysis of (5). The biological activities of metal complexes (such as antibacterial activity, which has been frequently reported recently) have been categorized into (3) and reported.

**(7)**  **Use of Schiff Bases in Materials Science or Engineering**

In addition to azo groups, imine groups are used in liquid crystals; some do not exhibit optical activity unless they are optically active [18]. One aspect of dealing with liquid crystals is the development of polymers into supramolecules. Porous solids include covalent organic frameworks. For the enantioselective adsorption of amino acids, a post-framework synthesis modification synthesis method was adopted [19]. Schiff bases are suitable for such synthesis because they can easily form imine groups and are easily hydrolyzed. Schiff base complexes of copper and rare earths have been reported to have the functions of magnetism (single molecule magnet), magneto-optical Faraday effect, and proton conduction. Proton conduction and single-molecule magnets alone are unrelated, but these are characterized by being optically active complexes of enantiomers [20] and homochiral [21] to exhibit the Faraday effect.

According to previous views of Schiff base compounds [22], these are most widely used as intermediates in organic synthesis, and applied as catalysts, pigments and dyes, polymer stabilizers, and so on. Many studies have been carried out on Schiff bases not only as organic compounds, but also as ligands for metal complexes. Needless to say, Schiff bases as organic ligands also played an important role in the development of inorganic coordination chemistry, and were an essential point in the development of bioinorganic chemistry and optical or magnetic materials in the solid state.

My (T.A.’s) recent review [23] that presents photofunctions of hybrid materials composed of Schiff base metal complexes and metal nanoparticles or semiconductors also dealt with ligand-induced chirality and applications. Here, again, the introduction of optical activity into Schiff bases at the molecular or material level (rather than at the crystalline level [24]) was occasionally observed. One reason for this is undoubtedly the ease of molecular design and organic synthesis, and the ease of binding to metal ions as organic ligands. In this way, this Special Issue aims to be a comprehensive, interdisciplinary review of Schiff base compounds, with an emphasis on the latest advances.
